# Supplementary material for: Optogenetic activation of heterotrimeric G-proteins by LOV2GIVe, a rationally engineered modular protein
Source: eLife. 2020 Sep 16;9:e60155. doi: 10.7554/eLife.60155 (PMC7515630; doi:10.7554/eLife.60155)
Supplement: Supplementary file 1. [file elife-60155-supp1.docx]

**SUPPLEMENTARY FILE 1**

>LOV2GIV (parental prototype)

TTGGCTACTACACTTGAACGTATTGAGAAGAACTTTGTCATTACTGACCCAAGGTTGCCAGATAATCCCATTATATTCGCGTCCGATAGTTTCTTGCAGTTGACAGAATATAGCCGTGAAGAAATTTTGGGAAGAAACTGCAGGTTTCTACAAGGTCCTGAAACTGATCGCGCGACAGTGAGAAAAATTAGAGATGCCATAGATAACCAAACAGAGGTCACTGTTCAGCTGATTAATTATACAAAGAGTGGTAAAAAGTTCTGGAACCTCTTTCACTTGCAGCCTATGCGAGATCAGAAGGGAGATGTCCAGTACTTTATTGGGGTTCAGAAGGATGGAACTGAGCATGTCCGAGATGCTGCCGAGAGAGAGGGAGTCATGGAGATTAAGAAAACTGCAGAAAATATTGATGAGGCGGCAAAAgaagttgttactctacaacagtttttggaagaaagcaataagcttacctcagtacagataaagtcctcaagttaa

ttggctactacacttgaacgtattgagaagaactttgtcattactgacccaaggttgcca

L A T T L E R I E K N F V I T D P R L P

gataatcccattatattcgcgtccgatagtttcttgcagttgacagaatatagccgtgaa

D N P I I F A S D S F L Q L T E Y S R E

gaaattttgggaagaaactgcaggtttctacaaggtcctgaaactgatcgcgcgacagtg

E I L G R N C R F L Q G P E T D R A T V

agaaaaattagagatgccatagataaccaaacagaggtcactgttcagctgattaattat

R K I R D A I D N Q T E V T V Q L I N Y

acaaagagtggtaaaaagttctggaacctctttcacttgcagcctatgcgagatcagaag

T K S G K K F W N L F H L Q P M R D Q K

ggagatgtccagtactttattggggttcagaaggatggaactgagcatgtccgagatgct

G D V Q Y F I G V Q K D G T E H V R D A

gccgagagagagggagtcatggagattaagaaaactgcagaaaatattgatgaggcggca

A E R E G V M E I K K T A E N I D E A A

aaagaagttgttactctacaacagtttttggaagaaagcaataagcttacctcagtacag

K E V V T L Q Q F L E E S N K L T S V Q

ataaagtcctcaagttaa

I K S S S -

>LOV2GIVe (optimized variant “e”)

TTGGCTACTACACTTGAACGTATTGAGAAGAACTTTGTCATTACTGACCCAAGGTTGCCAGATAATCCCATTATATTCGCGTCCGATAGTTTCTTGCAGTTGACAGAATATAGCCGTGAAGAAATTTTGGGAAGAAACTGCAGGTTTCTACAAGGTCCTGAAACTGATCGCGCGACAGTGAGAAAAATTAGAGATGCCATAGATAACCAAACAGAGGTCACTGTTCAGCTGATTAATTATACAAAGAGTGGTAAAAAGTTCTGGAACCTCTTTCACTTGCAGCCTATGCGAGATCAGAAGGGAGATGTCCAGTACTTTATTGGGGTTCAGAAGGATGGAACTGAGCATGTCCGAGATGCTGCCGAGAGAGAGGGAGTCATGGAGATTAAGAAAACTGCAGAAAATATTgaagttgttactctacaacagtttttggaagaaagcaataagcttacctcagtacagataaagtcctcaagttaa

ttggctactacacttgaacgtattgagaagaactttgtcattactgacccaaggttgcca

L A T T L E R I E K N F V I T D P R L P

gataatcccattatattcgcgtccgatagtttcttgcagttgacagaatatagccgtgaa

D N P I I F A S D S F L Q L T E Y S R E

gaaattttgggaagaaactgcaggtttctacaaggtcctgaaactgatcgcgcgacagtg

E I L G R N C R F L Q G P E T D R A T V

agaaaaattagagatgccatagataaccaaacagaggtcactgttcagctgattaattat

R K I R D A I D N Q T E V T V Q L I N Y

acaaagagtggtaaaaagttctggaacctctttcacttgcagcctatgcgagatcagaag

T K S G K K F W N L F H L Q P M R D Q K

ggagatgtccagtactttattggggttcagaaggatggaactgagcatgtccgagatgct

G D V Q Y F I G V Q K D G T E H V R D A

gccgagagagagggagtcatggagattaagaaaactgcagaaaatattgaagttgttact

A E R E G V M E I K K T A E N I E V V T

ctacaacagtttttggaagaaagcaataagcttacctcagtacagataaagtcctcaagt

L Q Q F L E E S N K L T S V Q I K S S S

taa

-
